# Supplementary material for: MYBL2 and ATM suppress replication stress in pluripotent stem cells
Source: EMBO Rep. 2021 Mar 28;22(5):e51120. doi: 10.15252/embr.202051120 (PMC8097389; doi:10.15252/embr.202051120)
Supplement: Supplementary file 2 — Expanded View Figures PDF [file EMBR-22-e51120-s001.pdf]

Expanded View Figures

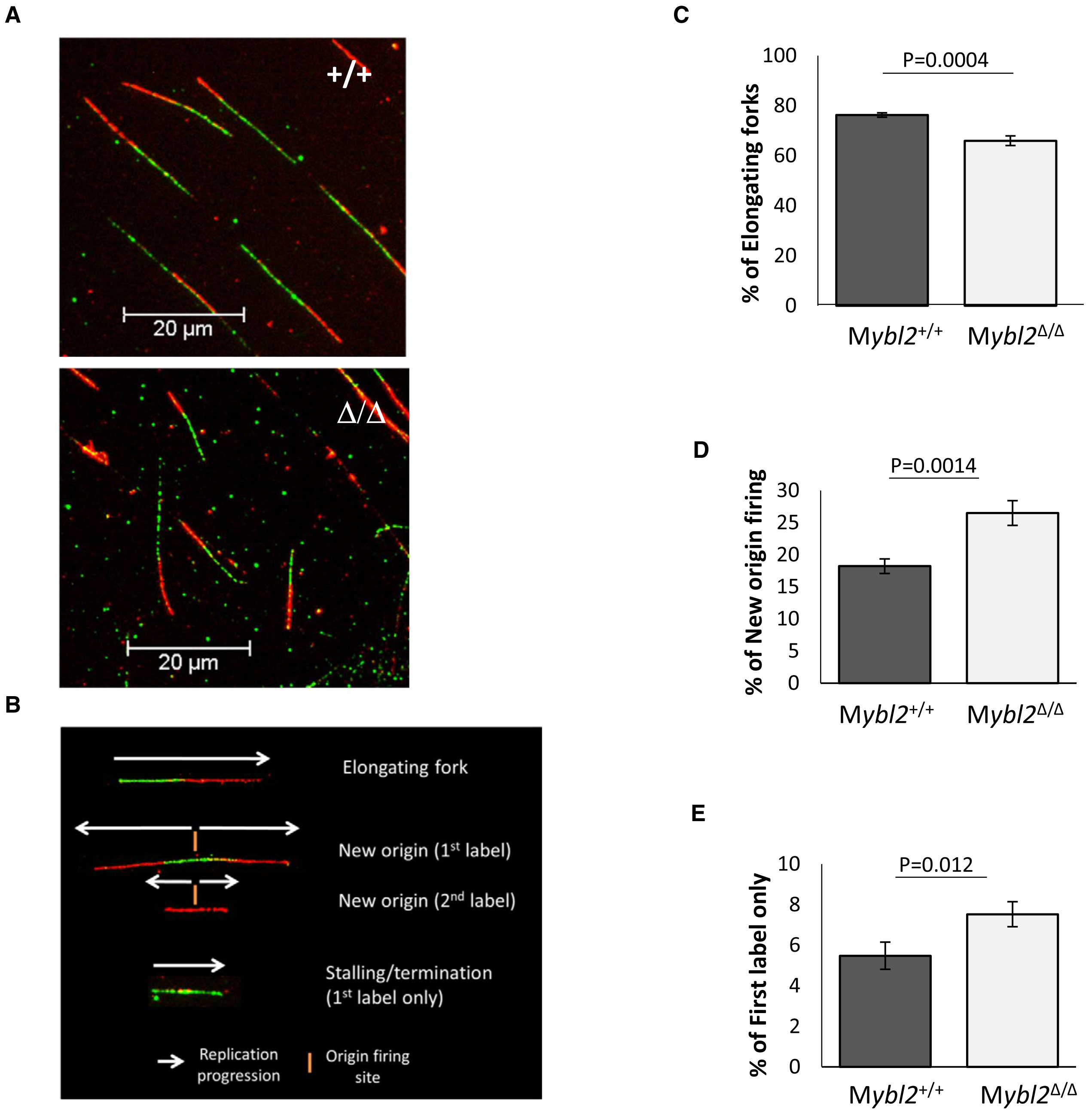

Figure EV1. Changes in the frequency of replication structures in *Mybl2*$^{\Delta/\Delta}$ *ESCs*.

A Representative images of replication tracks from *Mybl2*$^{+/+}$, *Mybl2*$^{\Delta/\Delta}$ *ESCs*.

B Representative images of new firing origins and unstable elongating forks scored.

C–E (C) Frequency of elongating forks, (D) frequency of new firing origins and (E) frequency of first label only slowing/stalling events in *Mybl2*$^{+/+}$, *Mybl2*$^{\Delta/\Delta}$ ESCs calculated as a per cent of all structures. Error bars represent SEM. Statistical analysis was carried out using two-tailed unpaired *t*-test. At least 1,000 total structures were counted per condition, from 6 independent repeats.

© 2021 The Authors

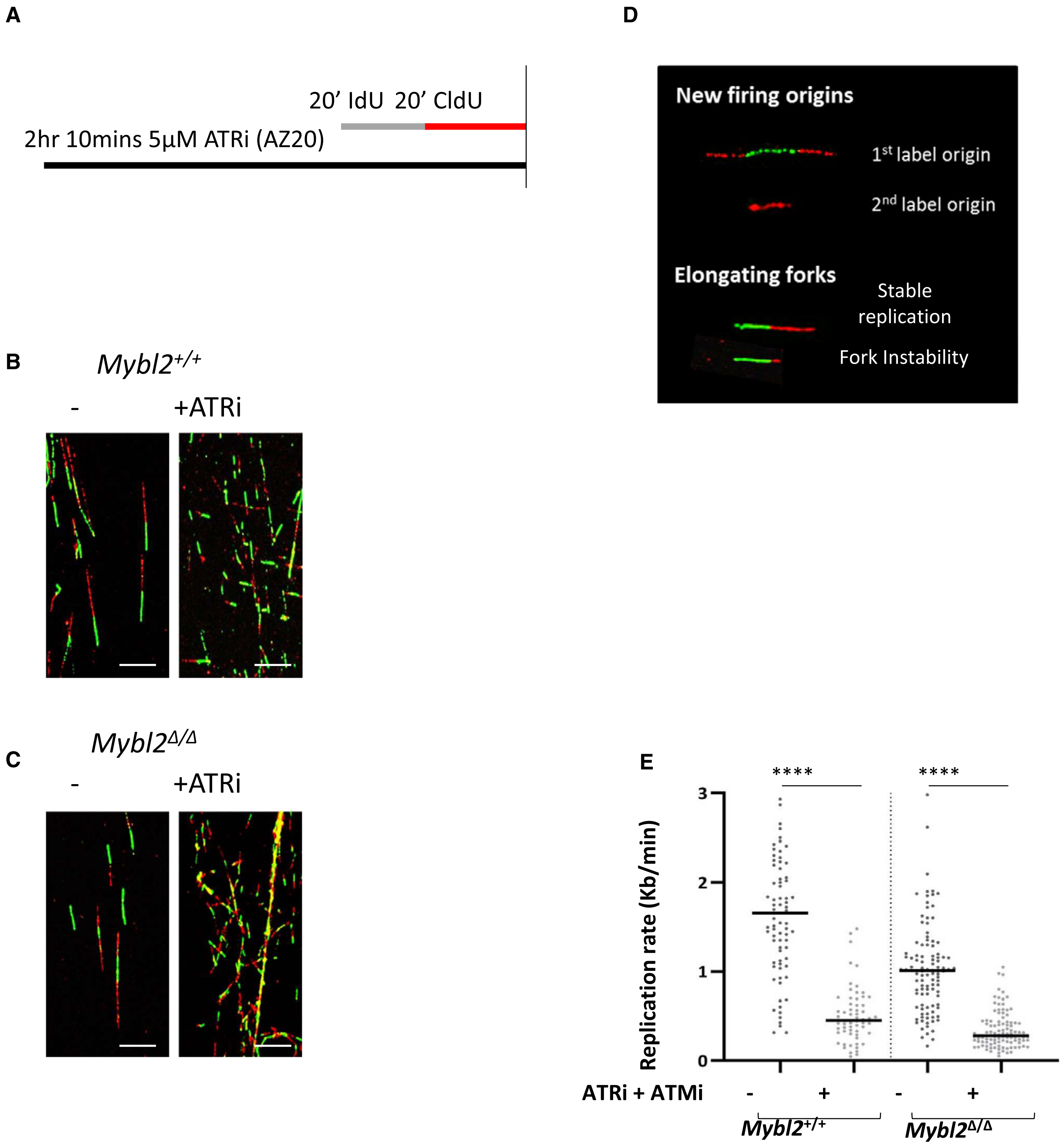

Figure EV2. Proficient ATR activation in MYBL2-ablated ESCs.

A Scheme of the procedure before DNA spreading. Cells were treated for 1.5 h with 5 μM ATR inhibitor (AZ20), before sequential addition of IdU and CldU for 20 min each.

B, C Representative images of replication tracks from *Mybl2*$^{+/+}$, *Mybl2*$^{\Delta/\Delta}$ ESCs treated and untreated with ATRi. Scale bar 10 μm.

D Representative images of new firing origins and instability of elongating forks scored.

E Distribution of replication fork rates for *Mybl2*$^{+/+}$, *Mybl2*$^{\Delta/\Delta}$ ESCs treated and untreated with ATR inhibitor AZ20 and ATMi KU66019. Statistical analysis was performed using the Mann–Whitney *U*-test (****$P < 0.0001$). $n = 2$ experimental replicates. A minimum of 100 replication forks was counted.

 © 2021 The Authors

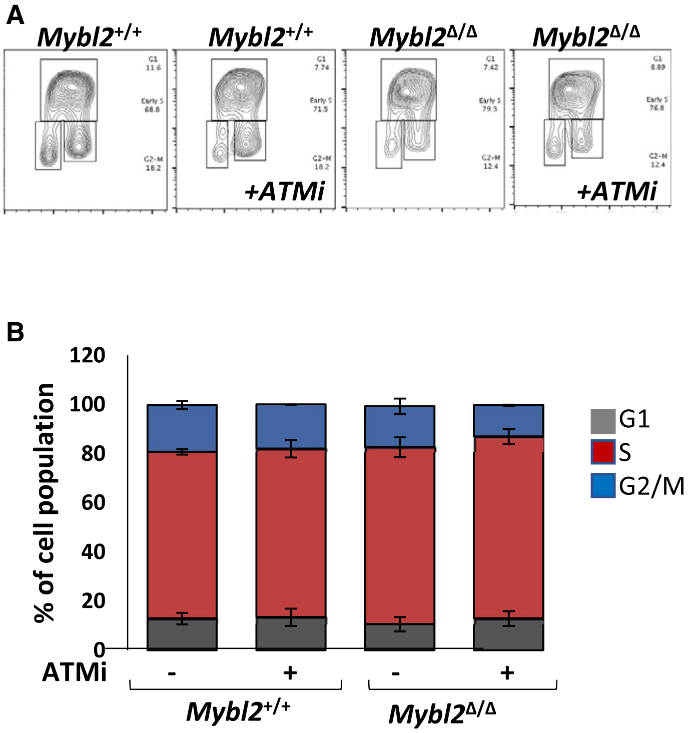

Figure EV3. MYBL2 deficiency and ATM inhibition do not alter the ESCs cell cycle.

A Representative contour plots.

B Frequency of cells on the different phases of the cell cycle.

Data information: *Mybl2*$^{+/+}$ and *Mybl2*$^{\Delta/\Delta}$ ESCs untreated and treated with ATM inhibitor (KU60019) for 2 h were given BrdU (25 μM) for the last hour of treatment. DNA synthesis and DNA content were assessed by flow cytometry. The proportion of the cells in the cell cycle was analysed using FlowJo (untreated $n = 4$; ATM inhibitor treated $n = 2$ independent experiments). Error bars represent SD.

Figure EV4. Inhibition of ATM and MYBL2 deficiency display similar number of replication factories and replication-associated genome instability.

A Scheme of the experimental design.

B Frequency of EdU-positive cells showing more than six 53BP1 foci. At least 150 cells were counted per group from three separate repeats. Error bars represent SEM. Statistical analysis using two-tailed unpaired *t*-test.

C Representative images for *Mybl2*$^{+/+}$ and *Mybl2*$^{\Delta/\Delta}$ ESCs untreated and treated with ATM inhibitor (KU60019) taken at 40× magnification. DNA labelling (blue) EdU-positive replicating cells (red) and 53BP1 (green). (Scale bar 10 μm). Lower row shows merge staining. CPT treatment was used as positive control.

D Representative rendering images of replication factories for the different genotypes and treatments after cells were cultured for 20 min in the presence of IdU. (Scale bar 5 μm).

© 2021 The Authors

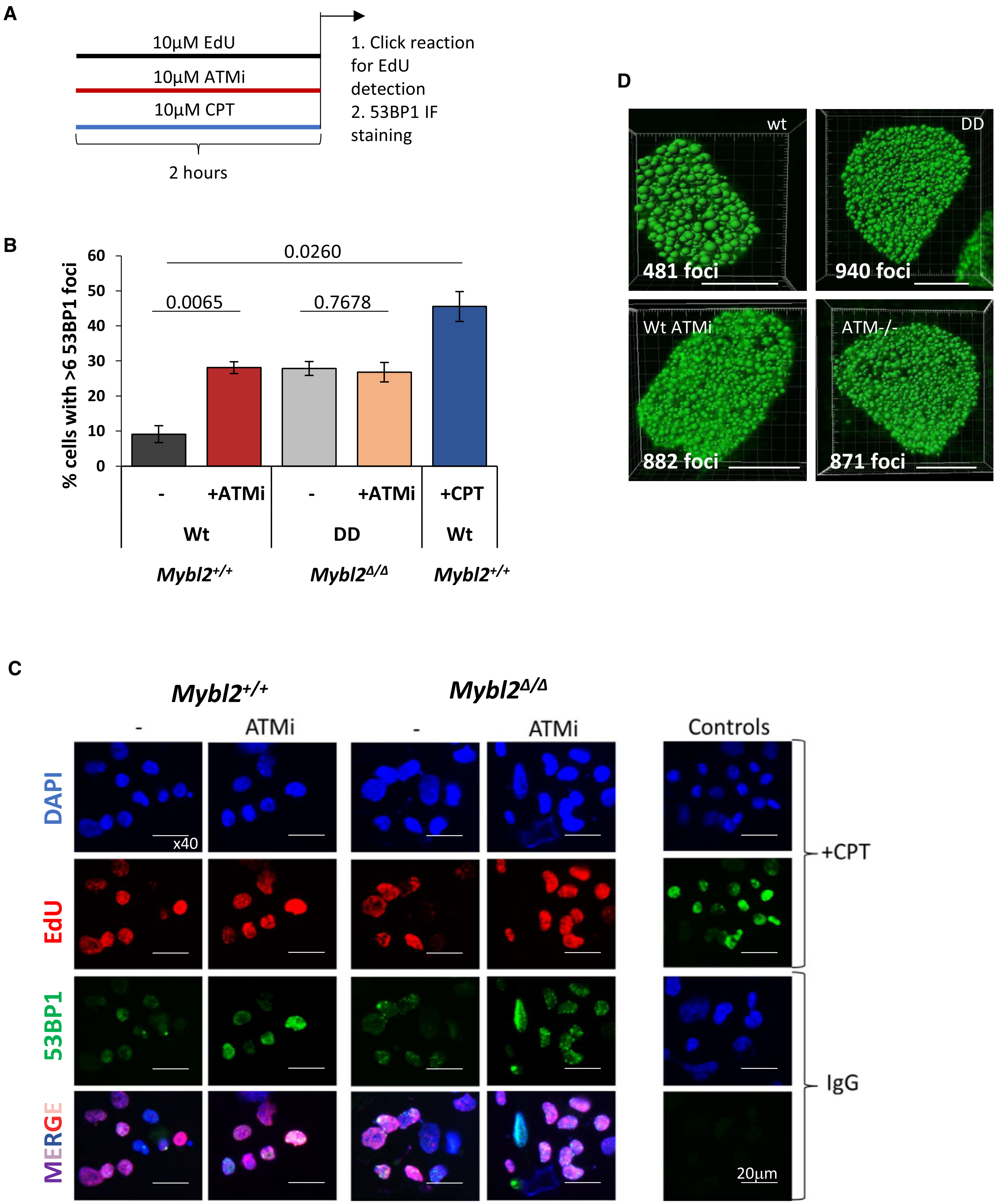

Figure EV4.

 © 2021 The Authors

Figure EV5. Replication speed phenotype in *Mybl2*$^{\Delta/\Delta}$ ESCs is partially rescued by CDK1 and CDK1/2 inhibition.

A Scheme of the aim and procedure before DNA spreading. Cells were treated for 1.5 h with CDK1 inhibitor (RO3306) or ATM inhibitor (KU60019) alone or in combination, before sequential addition of IdU and CldU for 20 min each.

B Frequency of new firing origins (relative to total structures counted) from *Mybl2*$^{+/+}$ and *Mybl2*$^{\Delta/\Delta}$ ESCs treated or not with the indicated inhibitors. At least 300 replication structures were counted per treatment. Error bars represent SEM. Statistical analysis using two-tailed unpaired *t*-test (ns = no significant).

C Replication rate (kb/min) of Mybl2$^{+/+}$ and Mybl2$^{\Delta/\Delta}$ ESCs treated with the indicated inhibitors. Statistical analysis was carried out using an unpaired Mann–Whitney *U*-test (****$P < 0.0001$). For non-ATMi treatments, at least 300 forks were scored from four independent experiments. A minimum of 130 forks was scored for ATM inhibitor-treated groups. From two independent experiments.

D Scheme of the aim and procedure before DNA spreading. Cells were treated for 1.5 h with CDK1/2 inhibitor III or ATM inhibitor (KU60019) alone or in combination, before sequential addition of IdU and CldU for 20 min each.

E Frequency of new firing origins (relative to total structures counted) from *Mybl2*$^{+/+}$ and *Mybl2*$^{\Delta/\Delta}$ ESCs treated or not with the indicated inhibitors. At least 200 replication structures were counted per treatment from three independent experiments. Error bars represent SEM. Statistical analysis using two-tailed unpaired *t*-test (*$P < 0.05$; **$P < 0.01$; ns = no significant).

F Replication rate (kb/min) of *Mybl2*$^{+/+}$ and *Mybl2*$^{\Delta/\Delta}$ ESCs treated with the indicated inhibitors. Statistical analysis was carried out using an unpaired Mann–Whitney *U*-test (***$P < 0.001$; ****$P < 0.0001$). A minimum of 130 forks was counted from three independent experiments. T.

G Frequency of new origins (relative to total structures counted) and replication rate (kb/min) of Mybl2$^{+/+}$ ESCs treated with 3 μM of CDK1/2 inhibitor III. ($n = 1$). A total of 100 forks were counted. Statistical analysis using two-tailed unpaired *t*-test.

© 2021 The Authors

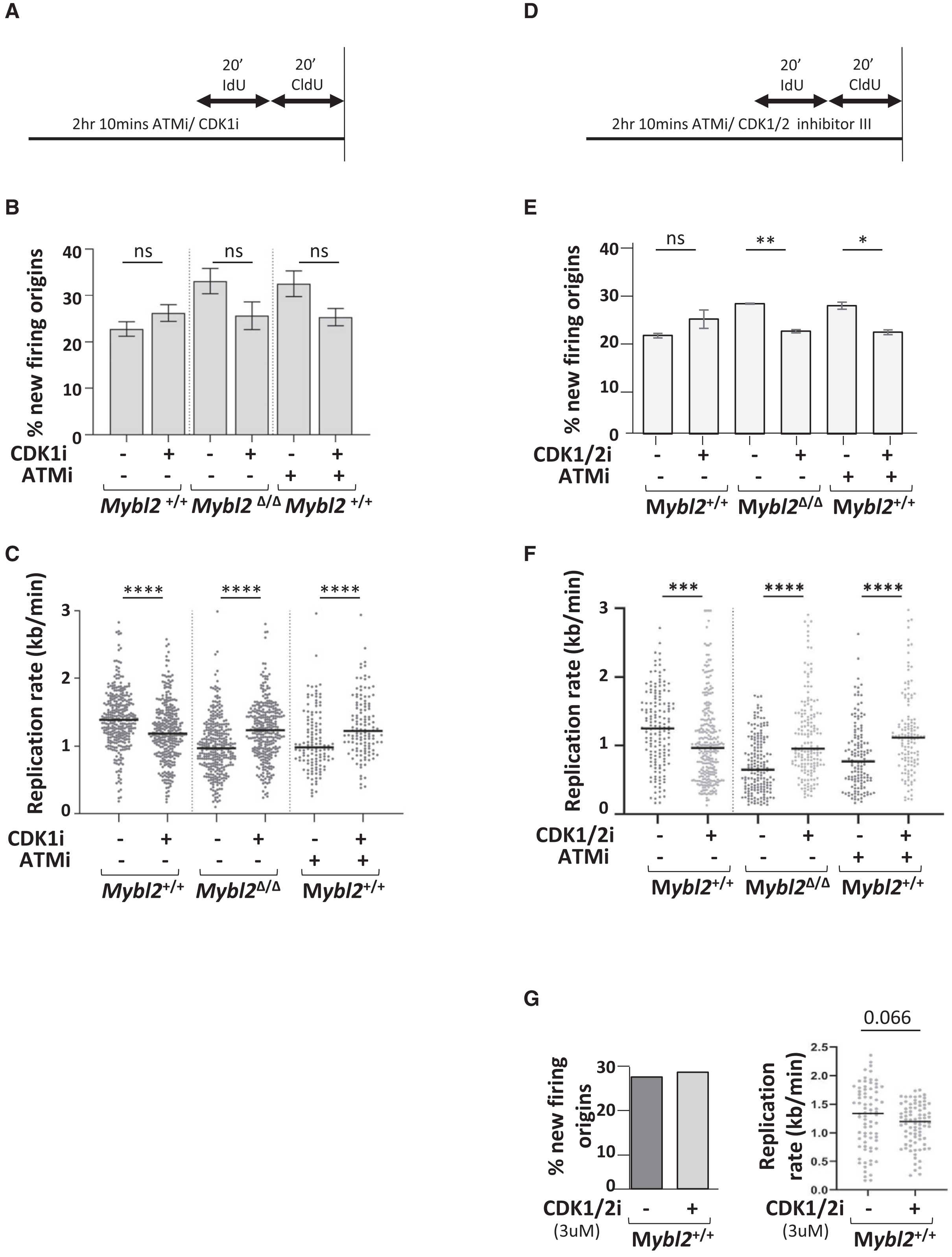

Figure EV5.

 © 2021 The Authors
